# Evaluation of the MTBDR*plus* 2.0 assay for the detection of multidrug resistance among persons with presumptive pulmonary TB in China

**DOI:** 10.1038/s41598-017-03473-7

**Published:** 2017-06-13

**Authors:** Yaoju Tan, Qiang Li, Qing Wang, Huiping Sun, Jin Chen, Xingshan Cai, Yinchai Yao, Xundi Bao, Chao Wang, Yuan Liu, Xia Wu, Yu Pang, Yanlin Zhao

**Affiliations:** 1 Department of Clinical Laboratory, Guangzhou Chest Hospital, Guangzhou, China; 2 National Tuberculosis Reference Laboratory, China Center for Disease Control and Prevention, Beijing, China; 3 Department of Clinical Laboratory, Anhui Chest Hospital, Hefei, China; 4 Department of Clinical Laboratory, Xi’an Chest Hospital, Xi’an, China; 5 Department of Clinical Laboratory, Shanghai Pulmonary Hospital, Shanghai, China; 6 Beijing Chest Hospital, Capital Medical University, Beijing, China

## Abstract

We have conducted a multicenter study of the diagnostic accuracy of the MTBDR*plus* 2.0 assay in compared with conventional and molecular reference standard in four tuberculosis (TB)-specialized hospitals of China. A total of 5038 patients were enrolled in this study. The overall sensitivity of the assay for the diagnosis of TB was 92.7% [1723/1858, 95% confidence interval (95% CI): 91.5–93.9]. In smear-positive/culture-positive cases the sensitivity was 97.7% (995/1018, 95% CI: 96.6–98.6), whereas in smear-negative/culture-positive cases it was 86.7% (728/840, 95% CI: 84.2–88.9). The agreement rate between MTBDR*plus* 2.0 and Xpert MTB/RIF was 97.7% (1015/1039, 95% CI: 96.6–98.5) for smear-positive cases and 97.0% (3682/3794, 95% CI: 96.5–97.6) for smear-negative cases. As compared with phenotypic drug susceptibility testing, the MTBDR*plus* 2.0 correctly identified 298 of 315 patients (94.6%, 95% CI: 91.5–96.8) with rifampicin-resistance. As noted previously, isoniazid resistance is associated with many different mutations and consequently the sensitivity compared to phenotypic testing was lower (81.0%, 95% CI: 76.8–84.7). In conclusion, this assay is a rapid, accurate test in terms of increased sensitivity for detecting smear-negative TB patients, as well as an alternative for detecting both RIF and INH resistance in persons with presumptive TB, whereas the absence of a mutation in the specimens must be interpreted cautiously.

## Introduction

Multidrug-resistant tuberculosis (MDR-TB), defined as resistance to at least isoniazid (INH) and rifampicin (RIF), poses as a major threat to global tuberculosis control^1–3^. According to the 2015 World Health Organization (WHO) report, there were an estimated 580,000 MDR /RIF-resistant tuberculosis (RR-TB) cases, and 250,000 deaths from MDR/RR-TB in 2015^4^. China, India and the Russian Federation carried the greatest estimated burden of MDR tuberculosis, accounting for 54% of the global cases^4, 5^. Despite the high incidence of MDR-TB occurring in high TB-burden countries, only about a quarter of MDR-TB cases were detected and reported in 2014, reflecting the real difficulties in the diagnosis of MDR-TB^6^.

Bacterial culture-based drug susceptibility testing (DST) is still the standard for detecting MDR-TB^7^. Due to the slow growth rate of *Mycobacterium tuberculosis* (MTB), these methods always require several weeks to generate available results^8^. This creates a diagnostic delay that increases morbidity and mortality for patients, and enhances transmission in the community^9^. In recent years, an increasing array of new tools for the diagnosis of multidrug resistance has been introduced to overcome the inherent delays due to the use of phenotypic DST methods^6^. In 2008, the GenoType MTBDR*plus* (Hain Lifescience, Germany) was approved by the WHO for the detection of MTB and for identifying rifampicin (RIF) and isoniazid (INH) resistance from cultured isolates and from smear-positive sputum samples^10, 11^. The Genotype MTBDR*plus* assay provided a rapid option for detecting multidrug resistance from smear-positive samples^11^, while its turnaround time was still no less than 10 days for smear-negative samples due to the requirement for prior bacterial culture. Another endorsed molecular test platform for the detection of MTB and drug-resistance to RIF is Xpert MTB/RIF (Cepheid, USA)^12^. This automated molecular assay can diagnose TB and RIF-resistant TB directly from sputum samples in 2 hours, which overcomes many of the current operational difficulties^13, 14^. Despite exhibiting high accuracy for the diagnosis of TB, an obvious shortcoming of Xpert is that it can only detect RIF resistance rather than multidrug resistance. Considering the limitations of current available kits, there is an urgent need to develop molecular diagnostic tools for the diagnosis of MDR-TB from both smear-positive and smear-negative samples.

Hain Lifescience has developed a new MTBDR*plus* 2.0 test with a higher analytical sensitivity when compared with the original MTBDR*plus* ^15, 16^, which allows this new version to be performed on both smear-positive and smear- negative clinical specimens. By combining an optimized PCR master mix and a redesigned amplification program the new test version has increased sensitivity. Here, we have conducted a multicenter study on the diagnostic accuracy of the MTBDR*plus* 2.0 test across four TB-specialized hospitals in China. Our aim was to assess the performance of this assay for detecting TB and MDR-TB patients from persons with presumptive TB as compared with the conventional and molecular reference standards.

## Results

### Patients

A total of 5038 patients were enrolled in this study. As shown in Table 1, 1078 (21.4%) had smear-positive/culture-positive tuberculosis; 955 (19.0%) had smear-negative/culture-positive tuberculosis; 1899 (37.6%) were defined as clinically diagnosed cases; and 1106 (22.0%) with no clinical evidence of tuberculosis, were classified as non-TB patients. Of the 5038 patients, 265 (5.3%) were excluded from the comparison between liquid culture and MTBDR*plus* 2.0 for a variety of reasons, including 86 culture contamination, 114 nontuberculous mycobacteria, and 65 smear-positive/culture-negative cases. In addition, all the cases with the valid results detected by both MTBDR*plus* 2.0 and Xpert MTB/RIF were included in the comparison between these two methods, and there were 4833 cases included in the final analysis.

**Table 1:** Distribution in final diagnostic category.

Final diagnostic category	Guangzhou	Shanghai	Anhui	Xi’an	Total
Smear and culture positive tuberculosis-no./total no.(%)	340/1296(26.2)	269/1253(21.5)	231/1216(19.0)	238/1273(18.7)	1078/5038(21.4)
Smear negative and culture positive tuberculosis-no./total no.(%)	187/1296(14.4)	215/1253(17.2)	287/1216(23.6)	266/1273(20.9)	955/5038(19.0)
Clinical tuberculosis-no./total no.(%)	297/1296(22.9)	419/1253(33.4)	594/1216(48.8)	589/1273(46.3)	1899/5038(37.6)
No. tuberculosis-no./total no.(%)	472/1296(36.4)	350/1253(27.9)	104/1216(8.6)	180/1273(14.1)	1106/5038(22.0)

### Performance for case detection

The performance of MTBDR*plus* 2.0 for the diagnosis of TB is shown in Table 2. The overall sensitivity of the assay was 92.7% (1723/1858, 95% confidence interval [CI], 91.5–93.9). We observed no significant variation in overall sensitivity across the four sites (*P* = 0.077). In smear-positive/culture-positive cases the sensitivity was 97.7% (995/1018, 95% CI, 96.6–98.6), whereas in smear-negative/culture-positive cases it was 86.7% (728/840, 95% CI, 84.2–88.9), which was significantly lower than that in the smear-positive/culture-positive group (*P* < 0.01). The estimated specificity for the diagnosis of TB was 94.5% for MTBDR*plus* 2.0 (2755/2915, 95% CI, 93.6–95.3). Statistical analysis revealed that MTBDR*plus* 2.0 exhibited excellent agreement with liquid culture for detecting TB cases, with a kappa value of 0.87.

**Table 2:** Performance of MTBDRplus 2.0 for detecting tuberculosis in clinical sputum samples.

Site	Culture				GeneXpert		
	Sensitivity			Specificity	Agreement		
	All culture-positive	Smear-positive and culture-positive	Smear-negative and culture-positive	No tuberculosis	Smear-positive	Smear-negative	Total
Guangzhou (%)	434/455(95.4)	315/316(99.7)	119/139(85.6)	684/728(94.0)	311/317(98.1)	857/875(97.9)	1168/1192(98.0)
95% CI	93.0–97.1	98.3–100.0	78.66–90.98	92.0–95.6	95.9–99.3	96.8–98.8	97.0–98.7
Shanghai (%)	380/410(92.7)	230/239(96.2)	150/171(87.7)	728/756(96.3)	252/262(96.2)	919/958(95.9)	1171/1220(96.0)
95% CI	89.7–95.0	93.0–98.3	83.0–93.1	94.7–97.5	93.1–98.2	94.5–97.1	94.7–97.0
Anhui (%)	462/505(91.5)	221/229(96.5)	241/276(87.3)	637/684(93.1)	224/228(99.6)	928/958(96.9)	1152/1186(97.1)
95% CI	88.7–93.8	93.2–98.5	82.8–91.0	91.0–94.9	95.6–99.5	95.6–97.9	96.0–98.0
Xi’an (%)	447/488(91.6)	229/234(97.9)	218/254(85.8)	706/747(94.5)	228/232(98.3)	978/1003(97.5)	1206/1235(97.7)
95% CI	88.8–93.9	95.1–99.3	80.9–89.9	92.6–96.0	95.6–99.5	96.3–98.4	96.6–98.4
Total (%)	1723/1858(92.7)	995/1018(97.7)	728/840(86.7)	2755/2915(94.5)	1015/1039(97.7)	3682/3794(97.0)	4697/4833(97.2)
95% CI	91.5–93.9	96.6–98.6	84.2–88.9	93.6–95.3	96.6–98.5	96.5–97.6	96.7–97.6

CI: confidence interval.

We also compared the agreement rate for detecting TB between MTBDR*plus* 2.0 and Xpert MTB/RIF. As shown in Table 2, the overall agreement rate between the two assays was 97.2% (4697/4833, 95% CI, 96.7–97.6), with a kappa value of 0.94. When separating participants into smear-positive and smear negative cases, the agreement rate was 97.7% (1015/1039, kappa value: 0.83) for smear-positive cases and 97.0% (3682/3794, kappa value: 0.92) for smear-negative cases. Statistical analysis revealed that there was no significant difference between smear-positive and smear-negative groups (*P* = 0.267).

### Performance for detecting drug resistance

Using the phenotypic liquid culture method, BACTEC MGIT960 DST (BD Diagnostics, USA) 315 samples were identified as RIF resistant. In contrast, MTBDR*plus* 2.0 identified 17 of these samples as susceptible to RIF, indicating an overall sensitivity of 94.6% (95% CI, 91.5–96.8) for detecting RIF resistance. For 17 of 315 patients for whom results on the MTBDR*plus* 2.0 were discrepant against phenotypic testing, 16 (94.1%) were confirmed to be genotypically resistant by sequencing of the *rpoB* gene. Interestingly, the results of RIF susceptibility determined by Xpert MTB/RIF among 16 false negative cases were susceptible. In addition, the overall specificity of MTBDR*plus* 2.0 for RIF susceptibility was 95.6% (95% CI, 94.4–96.7). We observed that 59 patients identified as RIF-resistant by MTBDR*plus* 2.0 showed phenotypic susceptibility to RIF (Table 3). Further DNA sequencing analysis revealed that 46 (78.0%) out of 59 cases harbored genotypic mutations within the *rpoB* gene, including 4 (6.8%) strains with mutation in codon 511, 2 (3.4%) in codon 513, 1 (1.7%) in codon 515, 4 (6.8%) in codon 516, 3 (5.1%) in codon 522, 9 (15.3%) in codon 526, 18 (30.5%) in codon 531 and 5 (8.5%) in codon 533. In contrast, no genotypic mutations were detected in a screen of the *rpoB* gene from other false positive 13 strains (22.0%).

**Table 3:** Performance of MTBDRplus assay for detecting rifampicin resistance.

Site	MTBDRplus	Culture		Total	Sensitivity (%, 95% CI)	Specificity (%, 95% CI)	Positive predictive value (%, 95% CI)	Negative predictive value (%, 95% CI)
		R	S					
Guangzhou	R	77	13	90	95.1 (87.8–98.6)	96.1 (93.4–97.9)	85.6 (76.6–92.1)	98.8 (96.9–99.7)
	S	4	319	323				
	Total	81	332	413				
Shanghai	R	82	18	100	98.8 (93.5–100.0)	93.6 (90.0–96.2)	82.0 (73.1–89.0)	99.6 (97.9–100.0)
	S	1	262	263				
	Total	83	280	363				
Anhui	R	70	13	83	94.6 (86.7–98.5)	96.6 (94.3–98.2)	84.3 (74.7–91.4)	98.9 (97.3–99.7)
	S	4	373	377				
	Total	74	386	460				
Xi’an	R	69	15	84	89.6 (80.6–95.4)	95.7 (93.0–97.6)	82.1 (72.3–89.7)	97.7 (95.4–99.0)
	S	8	334	342				
	Total	77	349	426				
Total	R	298	59	357	94.6 (91.5–96.8)	95.6 (94.4–96.7)	83.5 (79.2–87.2)	98.7 (97.9–99.2)
	S	17	1288	1305				
	Total	315	1347	1662				

The comparison between the MTBDR*plus* 2.0 test and MGIT DST showed an overall sensitivity of 81.0% (95% CI, 76.8–84.7) for the detection of INH resistance. The sensitivity of MTBDR*plus* 2.0 for the diagnosis of INH resistance exhibited significant heterogeneity among the four sites in this study, ranging from 74.4% in Xi’an to 94.7% in Anhui (*P* = 0.004). Among the 78 phenotypically resistant strains showing wild-type bands at the *inhA* and *katG* genes, sequencing revealed that 71 strains carried no mutations in these two genes, thus confirming the MTBDR*plus* results. We also calculated the specificity of MTBDR*plus* for detecting INH resistance, and it correctly determined susceptibility to INH in 1187 of 1224 cases, with an overall specificity of 97.0% (95% CI, 95.9–97.9) (Table 4). Out of 37 strains with potentially “false positive” results, the presence of nucleotide substitution was observed among 27 strains (72.9%, 27/37), including 22 (59.5%) strains harboring mutations in the promoter region of *inhA* and 5 (13.5%) in the *katG* gene.

**Table 4:** Performance of MTBDRplus assay for detecting isoniazid resistance.

Site	MTBDRplus	Culture		Total	Sensitivity (%, 95% CI)	Specificity (%, 95% CI)	Positive predictive value (%, 95% CI)	Negative predictive value (%, 95% CI)
		R	S					
Guangzhou	R	72	9	81	78.3 (68.4–86.2)	97.1 (94.5–98.7)	88.9 (80.0–94.8)	93.7 (90.5–96.1)
	S	20	299	319				
	Total	92	308	400				
Shanghai	R	92	7	99	81.4 (73.0–88.1)	97.1 (94.2–98.8)	92.9 (86.0–97.1)	91.9 (87.9–94.9)
	S	21	238	259				
	Total	113	245	358				
Anhui	R	72	14	86	94.7 (87.1–98.6)	96.2 (93.7–97.9)	83.7 (74.2–90.8)	98.9 (97.2–99.7)
	S	4	356	360				
	Total	76	370	446				
Xi’an	R	96	7	103	74.4 (66.0–81.7)	97.7 (95.3–99.1)	93.2 (86.5–97.2)	89.9 (86.1–93.0)
	S	33	294	327				
	Total	129	301	430				
Total	R	332	37	369	81.0 (76.8–84.7)	97.0 (95.9–97.9)	90.0 (86.4–92.8)	93.8 (92.4–95.1)
	S	78	1187	1265				
	Total	410	1224	1634				

## Discussion

The low detection rate of MDR-TB is one of the major challenges influencing the gap between diagnosis and treatment in China (WHO). There are several reasons responsible for this issue: (1) the implementation of rapid molecular assays for the detection of drug resistance is unsatisfactory in resource-poor settings with a high MDR-TB burden; (2) at present, all the molecular assays approved by the Chinese Food and Drug Administration are only approved for use on smear-positive clinical samples, including the endorsed Xpert MTB/RIF. Hence, more than 50% of MDR-TB cases may be missed due to lack of accessibility to molecular laboratory examinations for smear negative patients^17^. In this study, we first conducted a multicenter study to assess the diagnostic accuracy of the new version 2 of the GenoType MTBDR*plus* test for the detection of multidrug resistance among persons with presumptive TB. Our results suggest that the MTBDR*plus* 2.0 assay provides an accurate option for the diagnosis of MDR-TB among both smear-positive and smear-negative TB cases.

The MTBDR*plus* 2.0 assay outperformed smear microscopy because it detected a significant proportion of smear-negative TB cases. The proportion of rapidly diagnosed cases relative to smear microscopy significantly improved from 53.6% to 92.7%. According to our observations in this study, nearly one third of culture-positive patients were smear-negative. Considering that the four city hospitals in this study were top level TB specialized hospitals, most of the patients seeking care there were likely associated with more severe symptoms, thereby accounting for more smear-positive patients. Hence, there may be a higher proportion of smear-negative culture-positive patients in the county and prefectural level dispensaries. Consistent with our hypothesis, the national survey conducted in 2010 revealed that smear-negative patients contributed half of the bacteriologically positive tuberculosis burden in China in 2010^17^. Taking into account the high prevalence of smear-negative patients, there is an urgent need to scale up use of a rapid molecular assay, such as MTBDR*plus* 2.0, to improve the early detection of TB in smear-negative patients in China. In addition, we also found that the sensitivity (97.7%) of MTBDR*plus* 2.0 for detection of MTB in smear-positive patients was significantly higher than that (86.7%) for the smear-negative group, indicating that the bacterial load correlated strongly with test performance. Similar results were observed by Theron and colleagues, showing that the sensitivity of Xpert MTB/RIF in smear-negative TB was limited by bacterial load^9^. Sample concentration may serve as an option for improving the performance of the molecular assays. Based on our previous experience, sample concentration could increase the positivity rate of MTBDR*plus* in smear-positive sputum samples, especially for sputum at the smear scanty level. Further studies are required to evaluate the effectiveness of sample concentration for the detection of MTB in smear-negative clinical samples.

Secondly, the MTBDR*plus* 2.0 assay demonstrated a good performance across all four sites for the detection of resistance to RIF, which is consistent with numerouspublications^15, 18, 19^. In contrast, the sensitivity of the assay for detection of resistance to INH was moderate (81.0%). A recent meta-analysis showed that the sensitivity of MTBDR*plus* was 88.7% for the diagnosis of INH resistance for smear-positive TB patients, which was higher than in our findings^18^. However, several evaluations of new diagnostic tools in China revealed that the sensitivity for detecting resistance to INH was 80.3% for Genechip^8^ and 80.2% for MTBDRplus^20^, respectively, similar to our observations. In addition, a molecular epidemiological study by Pang and other colleagues found that the combination mutations in the *katG* gene and the promoter of *inhA* gene only identify less than 75% of INH-resistant isolates in the MDR population in China, whereas 5.1% of MDR isolates only harbored point mutations in the *oxyR-ahpC* region^21^. Thus, this lower sensitivity for INH resistance was likely influenced by the high prevalence of *oxyR-ahpC* region mutations in China, which are not detected by the MTBDR*plus* 2.0 assay. Similarly, our results exhibited diverse sensitivities across the four sites, which may be also attributed to the heterogeneity of INH-resistant isolates circulating in different local regions in China.

There were several obvious limitations of the MTBDR*plus* 2.0 assay. First, despite novel modifications introduced into the DNA extraction procedure, the MTBDR*plus* 2.0 assay also requires extensive manual work, and a relatively long turn-around time when compared with Xpert MTB/RIF. Second, the common mutations in the *oxyR-ahpC* region need to be included in the MTBDR*plus* 2.0 assay to improve the sensitivity for INH resistance, which would then be more suitable for use in China. Third, about 15% of smear negative culture positive TB patients could not be detected by the MTBDR*plus* 2.0 assay, which highlights the urgent need to optimize the procedures to improve the detection sensitivity. Nevertheless, MTBDR*plus* 2.0 assay provides an ideal solution to simultaneously rule out both RIF and INH resistance from clinical sputum samples. With the support of the Global Fund, 125 prefectural laboratories have been equipped with MTBDR detection equipments. Fortunately, the MTBDR assay platform is also suitable for use with the MTBDR*plus* 2.0 assay. Hence, it is easy to integrate this novel tool with the existing platform, which will provide a promising solution at the prefectural level.

To the best of our knowledge, this study is the first multicenter evaluation of the MTBDR*plus* 2.0 assay in a high TB prevalence setting. Our data reveal that this assay is an accurate rapid test in terms of increased sensitivity for detecting smear-negative TB patients. In addition, it established a diagnosis with excellent rule-out value for detecting both RIF and INH resistance from persons with presumptive TB, especially for regions with a high prevalence of mono RIF resistance, as occurs in China. Considering that a small portion of drug-resistant clinical specimens harbor no detectable mutations, especially for INH-resistant cases, the results of the molecular assay must be interpreted cautiously.

## Materials and Methods

### Study Population

From April 2014 through April 2015, we conducted this study at four TB specialized hospitals: Guangdong Chest Hospital, Shanghai Pulmonary Hospital, Xi’an Chest Hospital, and Anhui Chest Hospital. All patients with symptoms suggestive of pulmonary tuberculosis were consecutively enrolled in this evaluation. We collected sputum specimens of 1.5 mL each from patients. All patients gave written informed consent before they were included in this study. Ethical approval was granted by the Ethics Committee of the Chinese Center for Disease Control and Prevention. The methods were performed in accordance with the approved guidelines.

### TB case definitions

A person with presumptive TB patient was defined as someone presenting at the clinic with a cough of 2 weeks or more. Each person with presumptive TB was classified in one of three diagnostic categories: (1) confirmed TB patients: clinical TB symptoms with at least one sputum sample culture-positive for *M. tuberculosis*. The confirmed patients were further divided into smear-positive culture-positive patients and smear-negative culture-positive patients according to sputum smear results. (2) clinically diagnosed TB patients: clinical TB symptoms without any sputum sample culture-positive for *M. tuberculosis* plus a clinical-radiologic picture highly suggestive of TB (3) non-TB patients: no evidence of TB based on clinical examination.

### Laboratory methods

Direct smears from each sputum specimen were examined using Auramine O staining for acid fast bacilli (AFB) (National guidelines for TB laboratories, China Center for Disease Control and Prevention)^22^. The specimens were further digested with N-acetyl-L-cysteine and sodium hydroxide (NALC-NaOH) for 15 minutes. PBS buffer was added up to a total volume of 45 mL, and the suspension was centrifuged for 15 min at 3,000 × g. Following centrifugation, the supernatant was discarded and the sediment was suspended in 1.5 mL of PBS buffer. A0.5 mL portion of suspension was inoculated into a BACTEC MGIT tube (BD Microbiology Systems, USA); a second 0.5 mL of suspension was used to perform the MTBDR*plus* 2.0 test according to the manufacturer’s instructions; the final 0.5 mL of suspension was mixed with 1.5 mL of Xpert sample reagent for a 15-minute period at room temperature, then 2 mL of the inactivated material was transferred to a cartridge for GeneXpert MTB/RIF analysis (Cepheid, USA).

Positive cultures from specimens were preliminarily identified as *M. tuberculosis* complex with the MPT64 antigen kit (Genesis, Hangzhou, China)^23^. Indirect drug-susceptibility testing with the MGIT 960 SIRT was performed to detect the susceptibility of *M. tuberculosis* isolates to RIF and INH as per manufacturer’s instructions^24^. All the participating laboratory workers received standardized training, and were further approved by the National Reference Laboratory of Tuberculosis, China. The four clinical laboratories have participated in DST proficiency tests of the National TB Reference Laboratory since 2007.

### DNA Sequencing

Crude genomic DNA was extracted from the positive cultures as previously described^21^. Briefly, colonies were scraped from the surface of Lowenstein-Jensen medium and heated for 1 h at 95 °C in 500 μl Tris-EDTA (TE) buffer. Followed by centrifugation at 13,000 rpm for 2 min, the supernatant was used as a template for amplification and was sequenced for the corresponding gene fragments (*rpoB* for RIF resistance; *katG* and *inhA* promoter for INH resistance) by the Sanger method with the designed internal primers^21^. The PCR mixture was prepared in a final volume of 50 μL containing 25 μL 2×PCR Mixture (Genestar, Beijing, China), 2 μL of DNA template and 0.2 μM of each primer set. The PCR was carried out under the following conditions: initial denaturation at 94 °C for 5 min, and then 35 cycles of denaturation at 94 °C for 1 min, annealing at 58 °C for 1 min, and extension at 72 °C for 1 min, followed by a final extension at 72 °C for 10 min. The PCR products were sent to Tsingke Biotech Company for DNA sequencing service (Beijing, China). The sequencing results were analyzed by alignment with the corresponding reference strain (*M. tuberculosis* H37Rv; GenBank accession no. AL123456).

### Data analysis

The sensitivity, specificity, positive predictive value (PPV) and negative predictive value (NPV) were calculated to assess the performance of MTBDR*plus* 2.0 when using liquid culture and conventional phenotypic DST as reference standards, respectively. All of the data were evaluated by the National Reference Laboratory of Tuberculosis, then enrolled in SPSS15.0 as a data base and analyzed by the software. The kappa statistic was used to gauge the strength of agreement between MTBDR*plus* 2.0 assay and other methods. Values of the kappa coefficient higher than 0.75 indicated excellent agreement; values between 0.4 and 0.75 fair to good agreement; values lower than 0.4 poor agreement. In addition, the chi square test was performed to compare the performance of MTBDR*plus* 2.0 assay between different sample groups.
